# Poly[[tetra­deca­kis­(μ-propionato)hepta­barium] propionic acid monosolvate tetra­hydrate]

**DOI:** 10.1107/S2056989020000924

**Published:** 2020-01-31

**Authors:** Erika Samolová, Jan Fábry

**Affiliations:** a Inst. of Physics, v. v. i., Academy of Sciences of the Czech Republic, Na Slovance 2, 182 21 Praha 8, Czech Republic

**Keywords:** crystal structure, hydrogen bonding, metal–organic compounds, positional disorder, occupational disorder

## Abstract

The title compound, {[Ba_7_(C_3_H_5_O_2_)_14_]·0.946C_3_H_6_O_2_·4H_2_O}_*n*_, is represented by a metal–organic framework structure that is held together by Ba—O—Ba bonds, as well as by O—H⋯O hydrogen bonds of moderate strength. The structure comprises of four independent Ba^2+^ cations (one of which is situated on a twofold rotation axis), seven independent propionate and two independent water mol­ecules. The bond-valence sums of all the cations indicate a slight overbonding. There is also an occupationally, as well as a positionally disordered propionic acid mol­ecule present in the structure. Its occupation is slightly lower than the full occupation while the disordered mol­ecules occupy two positions related by a rotation about a twofold rotation axis. In addition, the methyl group in the symmetry-independent propionic acid mol­ecule is also disordered, and occupies two positions. Each propionic acid mol­ecule coordinates to just one cation from a pair of symmetry-equivalent Ba^2+^ sites and is simultaneously bonded by an O—H⋯O_propionate_ hydrogen bond. This means that on a microscopic scale, the coordination number of the corresponding Ba^2+^ site is either 9 or 10. The methyl as well as hy­droxy hydrogen atoms of the disordered propionic acid mol­ecule were not determined.

## Abstract 2

The structure of the title compound is held together by Ba—O—Ba bonds as well as by O—H⋯O hydrogen bonds of moderate strength. There is an occupationally as well as a positionally disordered mol­ecule of propionic acid in the structure. Its occupation deviates from a potentially full occupation while the disordered mol­ecules occupy two positions related by a twofold rotation axis.

## Chemical context   

A relatively low number of structurally determined metal propionates with divalent cations are known so far, as manifested by comparison of the numbers of propionates, acetates and formates with alkaline-earth cations which were retrieved from the Cambridge Structural Database (Groom *et al.*, 2016 ▸; version 5.40 from November 2018). Their numbers are 8, 60 and 70, respectively. One of the reasons for such a low number of determined structures might be associated with the tendency for difficult crystallization in case of some propionates. As an example of a difficult crystallization of a propionate salt from aqueous solution, Ca(propionate)_2_ and Cd(propionate)_2_ in a 2:1 molar ratio (Fábry, 2020 ▸) can be given.

Among the propionate salts, the most studied compounds are the isostructural salts Ca_2_Pb(propionate)_6_ and Ca_2_Sr(propionate)_6_. In the latter compounds, ferroelectric phases occur (see a short review by Nakamura & Deguchi, 1992 ▸). Structurally related Ca_2_Ba(propionate)_6_ shows inter­esting structural properties such as positional disorder of propionate chains in the room-temperature phase with symmetry *Fd* 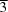 *m* (Stadnicka & Glazer, 1980 ▸). This disorder is a reason for diffuse streaks in the diffraction pattern, indicating correlated occurrence of the disordered propionate mol­ecules. The latter compound undergoes low-temperature phase transitions to phases with suggested ortho­rhom­bic symmetry (Gesi, 1993 ▸).
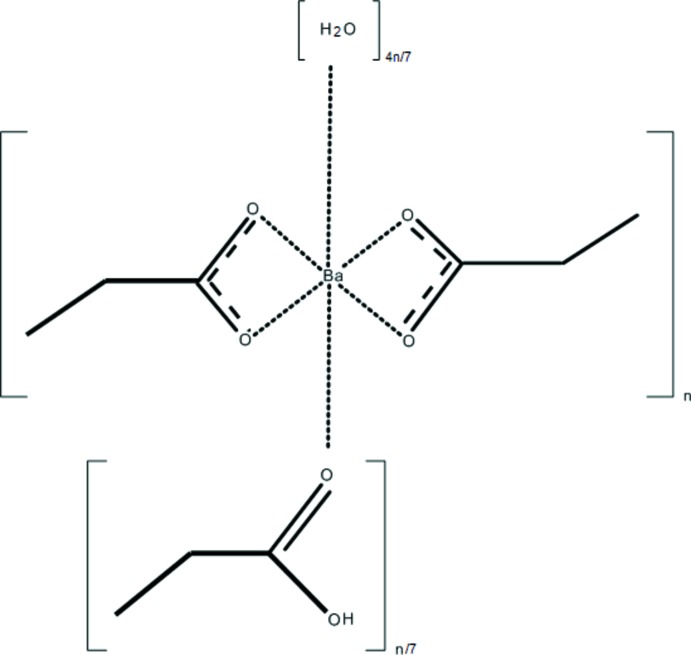


The title compound was prepared serendipitously. A few crystals of it were isolated from a batch of seemingly cubic crystals (they showed no extinction under polarized light) that grew from aqueous solutions of propionic acid (molar proportion > 30) with the amounts of BaCO_3_ and 4MgCO_3_·Mg(OH)_2_·4H_2_O in a molar ratio of 5:2; the pH of the solution was about 6. The motivation for the synthesis was a planned preparation of an analogue of Ca_2_Ba(propionate)_6_ where Ca^2+^ and Ba^2+^ are overbonded and slightly underbonded (Brese & O’Keeffe, 1991 ▸), respectively. For example, in the above-mentioned room-temperature phase of Ca_2_Ba(propionate)_6_, the bond-valence sums (Brese & O’Keeffe, 1991 ▸) of Ca^2+^ and Ba^2+^ amount to 2.78 (1) and 1.93 (1) valence units, respectively [see the refinement/model ‘A’ given in the article by Stadnicka & Glazer (1980 ▸) who discussed strong bonding of Ca^2+^ in this structure]. It was therefore hoped that a hypothetical structure ‘Ba_2_Mg(propionate)_6_’ might be isostructural to Ca_2_Ba(propionate)_6_ or related to it despite an expected lowering of the bond-valence sum by smaller Mg^2+^ cations. Indeed, alongside a few crystals of the title compound, cubic crystals were obtained, the structure determination of which is ongoing at present.

## Structural commentary   

**Figure 1:**
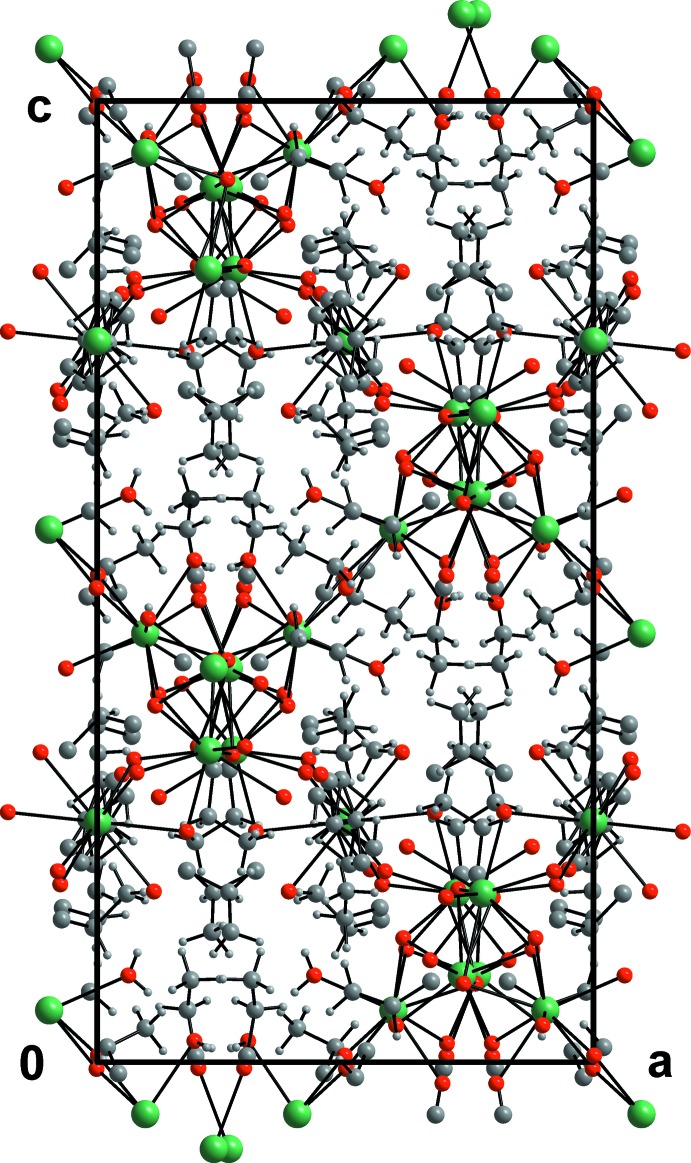
A view of the crystal structure along the *b* axis. Ba, O, C and water H atoms are shown as green, red, dark gray and tiny gray spheres, respectively.

**Figure 2:**
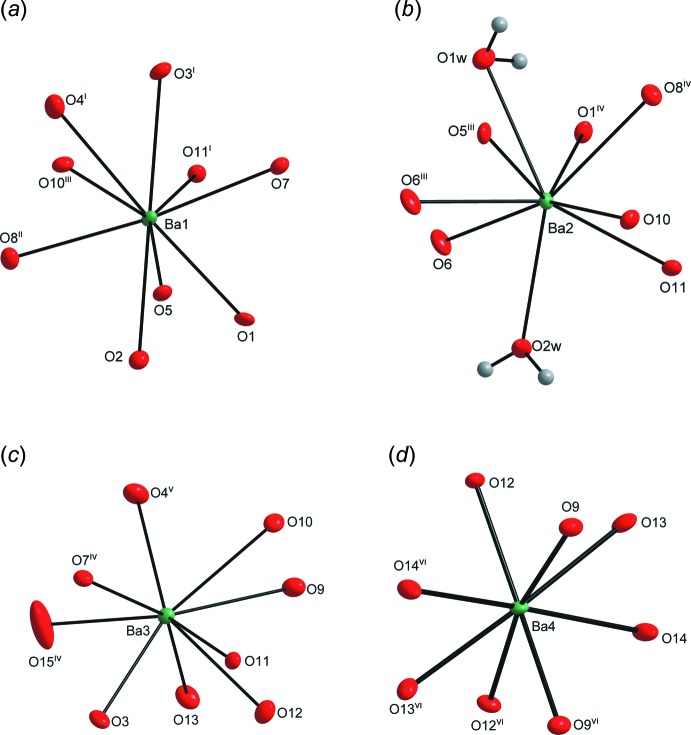
A view of the oxygen coordination around the cations, with displacement ellipsoids shown at the 50% probability level. (*a*) Ba1^2+^, (*b*) Ba2^2+^, (*c*) Ba3^2+^ and (*d*) Ba4^2+^ [Symmetry codes: (i) *x* − 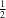, −*y* + 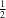, −*z* + 1; (ii) −*x* + 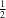, *y* + 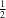, *z*; (iii) −*x* + 1, −*y* + 1, −*z* + 1; (iv) *x* + 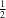, −*y* + 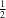, −*z* + 1; (v) *x* + 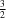, *y* + 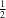, *z*; (vi) −*x* + 1, *y*, −*z* + 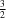].

**Figure 3:**
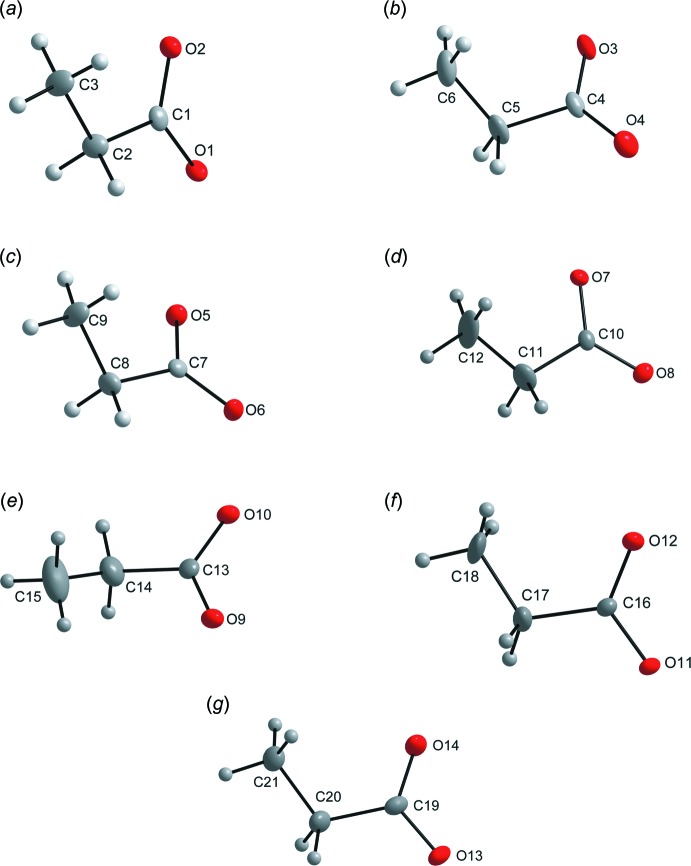
Mol­ecular structures of the propionate mol­ecules, with displacement ellipsoids shown at the 50% probability level. (*a*) mol­ecules with the carboxyl­ate atom C1, (*b*) mol­ecules with the carboxyl­ate atom C4, (*c*) mol­ecules with the carboxyl­ate atom C7, (*d*) mol­ecules with the carboxyl­ate atom C10, (*e*) mol­ecules with the carboxyl­ate atom C13, (*f*) mol­ecules with the carboxyl­ate atom C16 and (*g*) mol­ecules with the carboxyl­ate atom C19.

A view of the crystal structure is given in Fig. 1 ▸. There are four independent Ba^2+^ cations that are all coordinated by oxygen atoms stemming either from the carboxyl­ate or carb­oxy­lic groups or from water mol­ecules. The latter mol­ecules coordinate exclusively to Ba2^2+^. Ba3^2+^ is coordinated by the carb­oxy­lic group of an occupationally and positionally disordered propionic acid mol­ecule. Ba4^2+^ is situated on a twofold rotation axis, *i.e.* on the Wyckoff position *c*. An overview of the coordination environments around each of the Ba^2+^ cations is given in Table 1 ▸ with corresponding illustrations shown in Fig. 2 ▸ *a*–*d*. All Ba^2+^ cations are slightly overbonded (Table 1 ▸). Fig. 3 ▸ *a*–*g* shows all seven independent propionate mol­ecules coordinating the Ba^2+^ cations.

**Figure 4:**
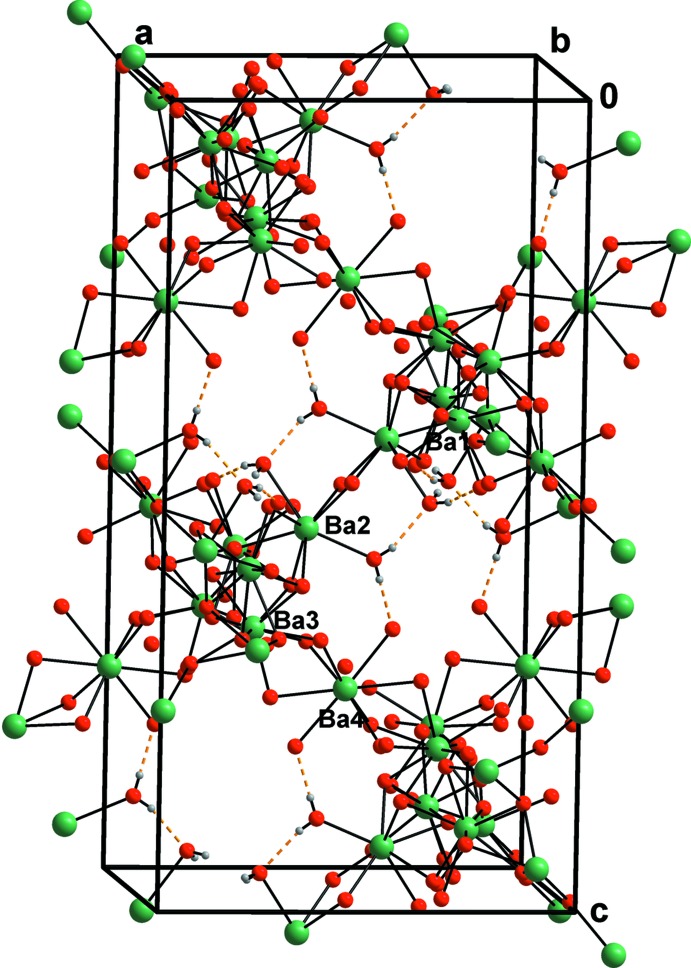
A view of the crystal structure excluding the propionate methyl­ene and methyl groups as well as the disordered propionic acid mol­ecule. Ba—O—Ba bonds and O—H⋯O bonds [except for O16⋯O4^vii^ (−*x* + 1, −*y*, −*z* + 1) where the bridging hydrogen atom was not found] are displayed. For colour codes, see caption for Fig. 1 ▸.

It can readily be seen from Fig. 1 ▸ that the cohesion within the crystal structure is mostly provided by a three-dimensional network of Ba—O—Ba bonds. This network is shown in more detail in Fig. 4 ▸, which also includes O—H⋯O hydrogen bonds of moderate strength (Gilli & Gilli, 2009 ▸). The corresponding donor groups are water mol­ecules while the acceptors are carboxyl­ate oxygen atoms. Numerical details of hydrogen-bonding inter­actions are provided in Table 2 ▸, excluding the O_propionic acid_—H⋯O_propionate_ hydrogen bond along O16⋯O4^vii^ [2.706 (13) Å; symmetry code: (vii) −*x* + 1, −*y*, −*z* + 1] that is donated by the free propionic acid mol­ecule. This mol­ecule is disordered over two positions related by (−*x* + 1, *y*, −*z* + 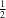) about a twofold rotation axis (Wyckoff position *c*). The low occupancy is probably the reason why the bridging hydrogen atom of the O16⋯O4^vii^ hydrogen bond could not be located in the difference electron density map. However, the angle C22—O16⋯O4^vii^, which measures 110.8 (8)°, is close to the tetra­hedral angle and is in agreement with the assumed presence of a hydrogen bond. The longer C22—O16 bond [1.303 (18) Å] in comparison with the C22—O15 bond [1.187 (12) Å)] indicates that the bridging hydrogen atom is attached to O16. Table 2 ▸ also lists a weak C—H⋯O inter­action between a methyl group and the carb­oxy­lic O atom of the propionic acid mol­ecule. The numerical parameters conform to the criteria for a weak hydrogen bond (Desiraju & Steiner, 1999 ▸).

**Figure 5:**
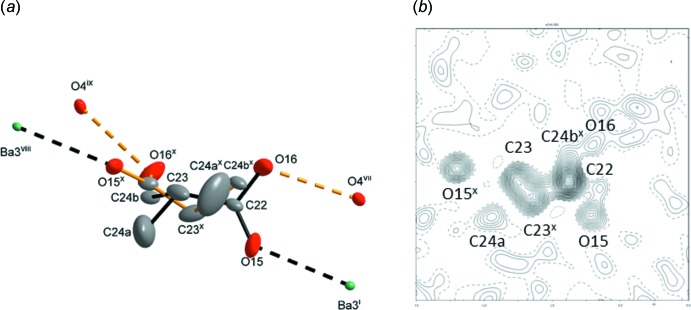
(*a*) A view of a disordered propionic acid mol­ecule with displacement ellipsoids shown at the 30% probability level. (*b*) Section of the difference electron density map (Petříček *et al.*, 2014 ▸) through the atoms C22, C23 and C24*b^x^*. This section shows the region of the disordered propionic acid mol­ecule in part. Increments of positive and negative contours are 0.01 and 0.05 e Å^−3^. [Symmetry codes: (i) *x* − 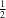, −*y* + 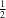, −*z* + 1; (vii) −*x* + 1, −*y*, −*z* + 1; (viii) −*x* + 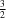, −*y* + 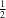, *z* − 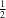; (ix) *x*, −*y*, *z* − 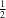]. The disordered atoms are related by a symmetry operation (Wyckoff position *c*) (*x*) −*x* + 1, *y*, −*z* + 1/29. For colours, see caption for Fig. 1 ▸.

Fig. 5 ▸ *a* shows a detailed view of the disordered propionic acid mol­ecule over two positions associated with the above-mentioned twofold rotation. The refined occupation of the mol­ecule of propionic acid converged to 0.473 (4) (full occupation of the site corresponds to 0.5). *SQUEEZE*, a functionality included in *PLATON* (Spek, 2015 ▸), yielded a value of 0.431. This means that the occupation of the disordered mol­ecule is not full; however, analysis of the bond-valence sum for Ba3^2+^ still points to a slight overbonding (Table 1 ▸) even without the presence of propionic acid. On a microscopic scale, the propionic acid mol­ecule is only bonded to one of the Ba3^2+^ cations from the pair of symmetry-equivalent cations (Ba3^i^ and Ba3^viii^; see Fig. 5 ▸ and the symmetry codes given therein) by the bond (O15—Ba3^i^, O15^x^—Ba3^viii^). At the same time, it forms the above mentioned O—H⋯O hydrogen bonds along O16⋯O4^vii^ and O16^x^⋯O4^ix^ [2.706 (13) Å]. In addition to the occupational disorder of the propionic acid mol­ecule, its methyl group was found to be disordered over two positions. One of these positions (the methyl C24*b* ^x^ atom) is very close to atom C22 (Fig. 5 ▸ *b*). The occupational parameters of the disordered methyl groups split into C24*a* and C24*b* converged to 0.30 (2) and 0.17 (1); methyl hydrogen atoms were not found. The displacement parameters of the methyl group C24*a* (Fig. 5 ▸ *a*) are quite large and indicate an intense libration. The displacement parameter of C24*b* was constrained to that of C22 (Fig. 5 ▸ *a*).

**Figure 6:**
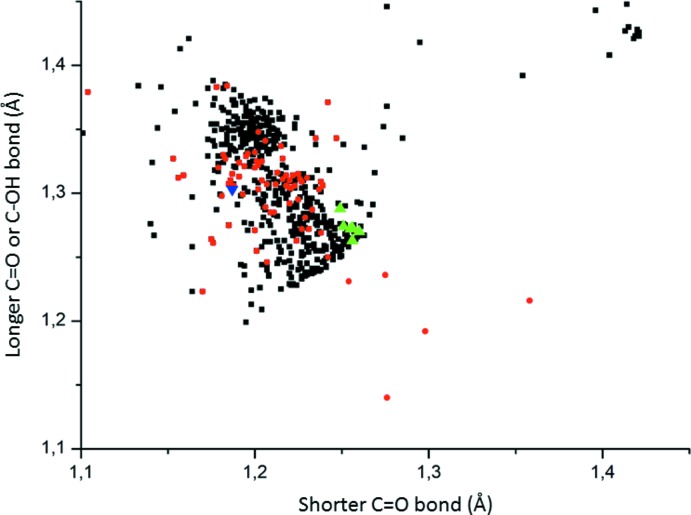
Scattergram of the distances for the shorter and the longer C=O bonds in the carboxyl­ate groups in propionates (black squares) as well as of C=O bonds and C—OH bonds in propionic acid mol­ecules (red circles). The corresponding values for the propionates and the propionic acid mol­ecule present in the title structure are shown as green and blue triangles, respectively.

Reported structures comprising propionate anions and/or propionic acid mol­ecules were retrieved from the Cambridge Structural Database (Groom *et al.*, 2016 ▸; version 5.40 from November 2018). Fig. 6 ▸ shows a scattergram of the shorter C—O (or C=O) and longer C—O (or C—OH) distances in the carboxyl­ate or carb­oxy­lic group, respectively. Corresponding distances in the title structure are normal although those pertinent to the carboxyl­ates are on the verge of the region where both C—O distances are about the same. Inter­estingly, there is no large difference between these parameters in the carboxyl­ate (black squares) and the carb­oxy­lic groups (red circles) in the propionate or propionic acid mol­ecules, respectively. There seem to be a clustering of points at about 1.21 and 1.35 Å, which manifest different bonding types in these mol­ecules.

## Synthesis and crystallization   

1 g of BaCO_3_ and 0.95 g of basic magnesium carbonate [Aldrich, product number 13118, the powder diagram of which corresponded best to that of the powder diffraction file 01-070-0361 of PDF-4 (Inter­national Centre for Diffraction Data, 2019 ▸)], *i.e.* 4MgCO_3_·Mg(OH)_2_·4H_2_O], were dissolved in an aqueous solution of 2.28 g of propionic acid. These masses correspond to molar ratios of 5:2:30. The majority of the solid dissolved in the acid solution and a few ml of propionic acid (100%) were added to the solution, maintaining its pH between 6 and 7. The solution was then filtered through a sintered disk. The filtrate was concentrated by evaporation at 323 K until colourless crystals appeared. A prevalent majority of the crystals were of cubic form with a typical size of 1 mm. Under a polarizating microscope, these crystals did not show extinction, *i.e.* they were optically isotropic. However, among these crystals a few crystals that showed extinction were found. They were isolated and one of them was chosen for single crystal X-ray structure determination.

## Structure determination and refinement   

Crystal data, data collection and structure refinement details are summarized in Table 3 ▸.

The structure can be divided into a non-disordered part composed of the Ba^2+^ cations, propionate anions and water mol­ecules, and the disordered mol­ecule of propionic acid. The refinement of the non-disordered structure part was straightforward, with methyl­ene hydrogen atoms calculated and their parameters constrained to C—H = 0.99 Å and *U*_iso_(H) = 1.2*U*_eq_(C). The methyl hydrogen atoms of the propionate mol­ecules were discernible in the difference electron density map. They were constrained with C—H = 0.98 Å and *U*_iso_(H) = 1.5*U*_eq_(C). The water hydrogen atoms were also discernible in the difference electron density map. Their positional parameters were restrained in such a way that O—H distances were set to 0.82 (1) Å, with *U*_iso_(H) = 1.5*U*_eq_(O). The residual maxima in the difference electron density map after the refinement of the non-disordered part of the structure conformed to the expected shape of the non-hydrogen atoms of a propionic acid mol­ecule (see Fig. 5 ▸ *a*,*b*). The functionality of *SQUEEZE* included in *PLATON* (Spek, 2015 ▸) indicated 138 electrons corresponding to the symmetry-related regions with the disordered mol­ecule present in the unit cell. Since a propionic acid mol­ecule has 40 electrons, the expected occupational parameter for the disordered mol­ecule is 138/160 = 0.8625 or 0.4313 for the occupancy considering the special position (twofold rotation axis) in its vicinity. The value of the expected occupancy is in fair agreement with the refined value of 0.473 (4) for the mol­ecule of propionic acid where four hydrogen atoms remained undetermined (the methyl as well as the hy­droxy hydrogen atoms). This disorder results in a statistical distribution of the mol­ecule about the twofold rotation axis, indicating that vacancies without the mol­ecule of propionic acid are likely to be present in the crystal structure. Reliability factors of a trial refinement with assumed full occupation of the disordered mol­ecule converged with neglig­ibly worse values and are collated in the refine_special_details section of the CIF. The respective electron densities of the peaks that were assigned to the atoms O15, O16, C22, C23 and C24*a* are 1.22, 0.97, 0.96, 0.82 and 0.31 e^−^ Å^−3^. The independently refined occupational parameters of the atoms of the disordered mol­ecule converged to the following values: O15: 0.410 (7); O16: 0.362 (7); C22: 0.571 (11); C23: 0.391 (9); C24: 0.184 (12), pointing to another type of occupational disorder, in particular regarding the distribution of the methyl group, which may partly overlap with atom C22 (Fig. 5 ▸ *b*). Treatment of these atoms after localization of all non-hydrogen atoms of the disordered propionic acid mol­ecule is described in detail in the refine_special_details section of the CIF.

43 reflections were discarded from the refinement because |*I*_obs −_ *I*_calc_|/*σ*(*I*_obs_) > 10. They are listed in the refine_special_details section of the CIF, together with the results of an alternative refinement with *SHELXL* (Sheldrick, 2015*b* ▸) where the contributions of the disordered propionic acid mol­ecule were removed using the *SQUEEZE* option in *PLATON* (Spek, 2015 ▸).

## Supplementary Material

Crystal structure: contains datablock(s) global, I. DOI: 10.1107/S2056989020000924/wm5531sup1.cif

Structure factors: contains datablock(s) I. DOI: 10.1107/S2056989020000924/wm5531Isup2.hkl

Supporting information file. DOI: 10.1107/S2056989020000924/wm5531Isup3.smi

Supporting information file. DOI: 10.1107/S2056989020000924/wm5531Isup4.cml

CCDC reference: 1979831

Additional supporting information:  crystallographic information; 3D view; checkCIF report

## Figures and Tables

**Table 1:** Bonding properties of Ba^2+^ cations^*a*^ in the title structure

Atom	Coordination number	*d*_min_(Ba—O) (Å)	*d*_max_(Ba—O) (Å)	Bond valence sum (v.u.)^*a*^
Ba1	9	2.666 (2)	2.923 (2)	2.191 (5)
Ba2	9	2.685 (2)	2.940 (2)	2.286 (5)
Ba3^*b*^	9.473	2.673 (2)	3.084 (2)	2.248 (5)
Ba3^*c*^	9.500	2.673 (2)	3.084 (2)	2.255 (5)
Ba3^*d*^	9	2.673 (2)	3.084 (2)	2.138 (5)
Ba3^*e*^	10	2.673 (2)	3.084 (2)	2.372 (6)
Ba4	8	2.670 (2)	2.868 (2)	2.204 (5)

Notes: (*a*) Calculation with the parameters of Brese & O’Keeffe (1991 ▸); (*b*) consideration of a disordered propionic acid mol­ecule with refined occupancy = 0.473 (4); (*c*) consideration of a disordered propionic acid mol­ecule with 0.5 occupancy; (*d*) excluding the disordered propionic acid mol­ecule; (*e*) local full occupation.

**Table 2:** Hydrogen-bond geometry (Å, °) The hydrogen bond O16⋯O4^vii^ is missing from this table because of the undetermined position of the bridging hydrogen atom.

*D*—H⋯*A*	*D*—H	H⋯*A*	*D*⋯*A*	*D*—H⋯*A*
O1*w*—H1*O*1*w*⋯O1^iv^	0.82 (3)	2.25 (3)	2.957 (3)	145 (4)
O1*w*—H2*O*1*w*⋯O2^iii^	0.82 (3)	2.00 (3)	2.813 (3)	171 (3)
O2*w*—H1*O*2*w*⋯O1*w* ^iii^	0.82 (3)	2.15 (3)	2.963 (4)	172 (4)
O2*w*—H2*O*2*w*⋯O14^vi^	0.81 (3)	2.01 (3)	2.807 (3)	164 (4)
C17—H1*c*17⋯O16^viii^	0.99	2.43	2.989 (15)	115.22

Symmetry codes: (iii) −*x* + 1, −*y* + 1, −*z* + 1; (iv) *x* + 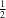, −*y* + 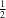, −*z* + 1; (vi) −*x* + 1, *y*, −*z* + 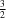; (vii) −*x* + 1, −*y*, −*z* + 1.

**Table 3:** Experimental details

Crystal data	
Chemical formula	[Ba_7_(C_3_H_5_O_2_)_14_]·0.946C_3_H_6_O_2_·4H_2_O
*M*_r_	2126.4
Crystal system, space group	Orthorhombic, *P* *b* *c* *n*
Temperature (K)	95
*a*, *b*, *c* (Å)	15.7831 (2), 14.0136 (2), 30.5583 (3)
*V* (Å^3^)	6758.83 (15)
*Z*	4
Radiation type	Mo *K*α
μ (mm^−1^)	4.10
Crystal size (mm)	0.22 × 0.12 × 0.10

Data collection	
Diffractometer	Rigaku Oxford Diffraction SuperNova, Dual, Cu at home/near, AtlasS2
Absorption correction	Multi-scan (*CrysAlis PRO*; Rigaku OD, 2019 ▸)
*T*_min_, *T*_max_	0.568, 0.656
No. of measured, independent and observed [*I* > 3σ(*I*)] reflections	112254, 8981, 8215
*R*_int_	0.043
(sin θ/λ)_max_ (Å^−1^)	0.696

Refinement	
*R*[*I* > 3σ(*I*)], *wR*(*F*), *S*	0.028, 0.071, 1.92
No. of reflections	8981
No. of parameters	428
No. of restraints	7
H-atom treatment	H atoms treated by a mixture of independent and constrained refinement
Δρ_max_, Δρ_min_ (e Å^−3^)	1.14, −0.72

Computer programs: *CrysAlis PRO* (Rigaku OD, 2019 ▸), *SHELXT* (Sheldrick, 2015*a* ▸), *JANA2006* (Petříček *et al.*, 2014 ▸), *DIAMOND* (Brandenburg, 2015 ▸), *Origin* (Origin, 2000 ▸) and *JANA2006* (Petříček *et al.*, 2014 ▸).

## supplementary crystallographic information

### Crystal data

**Table:**

[Ba_7_(C_3_H_5_O_2_)_14_]·0.946C_3_H_6_O_2_·4H_2_O	*F*(000) = 4063.4
*M**_r_* = 2126.4	*D*_x_ = 2.090 Mg m^−^^3^
Orthorhombic, *P**b**c**n*	Mo *K*α radiation, λ = 0.71073 Å
Hall symbol: -P 2n 2ab	Cell parameters from 70680 reflections
*a* = 15.7831 (2) Å	θ = 2.8–29.6°
*b* = 14.0136 (2) Å	µ = 4.10 mm^−^^1^
*c* = 30.5583 (3) Å	*T* = 95 K
*V* = 6758.83 (15) Å^3^	Prism, colourless
*Z* = 4	0.22 × 0.12 × 0.10 mm

### Data collection

**Table:**

Rigaku Oxford Diffraction SuperNova, Dual, Cu at home/near, AtlasS2 diffractometer	8981 independent reflections
Radiation source: X-ray tube	8215 reflections with *I* > 3σ(*I*)
Mirror monochromator	*R*_int_ = 0.043
Detector resolution: 5.2027 pixels mm^-1^	θ_max_ = 29.6°, θ_min_ = 2.4°
ω scans	*h* = −19→21
Absorption correction: multi-scan (CrysAlisPro; Rigaku OD, 2019)	*k* = −18→18
*T*_min_ = 0.568, *T*_max_ = 0.656	*l* = −40→41
112254 measured reflections	

### Refinement

**Table:**

Refinement on *F*^2^	196 constraints
*R*[*F* > 3σ(*F*)] = 0.028for R[I>3σ (I)]	H atoms treated by a mixture of independent and constrained refinement
*wR*(*F*) = 0.071	Weighting scheme based on measured s.u.'s *w* = 1/(σ^2^(*I*) + 0.0004*I*^2^)
*S* = 1.92	(Δ/σ)_max_ < 0.001
8981 reflections	Δρ_max_ = 1.14 e Å^−^^3^
428 parameters	Δρ_min_ = −0.72 e Å^−^^3^
7 restraints	

### Special details

**Table:**

Refinement. 1) Reliability factors considering full occupation of the disordered propionic acid molecule: _refine_ls_R_factor_gt (0.0277), _refine_ls_wR_factor_gt (0.0698), _refine_ls_R_factor_all (0.0324), _refine_ls_wR_factor_ref (0.0712), _refine_ls_goodness_of_fit_ref (1.93), _refine_ls_goodness_of_fit_gt (1.98).2) Details of the disorder in the proprionic acid molecule: The overall occupational parameter of the disordered propionic acid molecule was determined by refinement of the molecular part comprising of the atoms O15, O16 and C23 which seemed to be the ones least-affected by disorder or overlapping. This refined value has then been used as a value to which the sum of partial occupational parameters of the methyl atoms C24a and C24b should equal while refining the occupational parameter of C24b. The displacement parameter of C24b due to its proximity was supposed to be equal to that of C22 which was refined.The positions of the methylene hydrogen atoms of C23a and C23b H1C23a, H2C23a; H1C23b, H2C23b) were calculated, with occupational parameters constrained to be equal to the occupational parameters of C24a and C24b, respectively, and with C—H = 0.99Å, *U*_iso_(H) = 1.2*U*_eq_(C). The distance C22—C23 was restrained to 1.52 (1) Å while the distances C23—C24a and C23—C24b were restrained to 1.50 (1) Å. 3) 43 diffractions with (Iobs-Icalc)/sigma(w)>10 were discarded from the refinement:0 8 0; 6 4 1; 7 7 2; 1 8 2; 5 8 2; 6 8 2; 7 8 2; 2 10 2; 7 4 3; 8 1 4; 4 10 4; 3 4 5; 7 4 5; 6 6 6; 1 10 6; 5 10 6; 6 3 7; 3 4 7; 6 8 8; 0 10 8; 6 2 9; 1 3 9; 3 4 9; 6 6 9; 7 10 10; 5 1 11; 7 1 2; 3 3 12; 1 1 13; 5 1 13; 3 4 13; 4 5 13; 1 2 15; 1 6 15; 4 1 16; 0 2 17; 0 6 17; 1 1 19; 1 2 19; 3 4 21; 1 4 22; 1 1 25; 0 2 25.4) An alternative refinement of the structure with the disordered propionic acid molecule being removed was carried out with SHELXL (Sheldrick, 2015*b*) using the *SQUEEZE* option in *PLATON* (Spek, 2015). The refinement converged with the folowing reliability factors: _refine_ls_R_factor_all = 0.0308, _refine_ls_R_factor_gt = 0.0280, _refine_ls_wR_factor_ref = 0.0584, _refine_ls_wR_factor_gt = 0.0577, 385 parameters, 9024 diffractions, 4 restraints, condition for the observed diffractions I_obs_>2σ(I_obs_).These values are slightly better than those obtained from the refinement with *JANA*2006 (Petříček *et al.*, 2014) with the same conditions for the observed diffractions I_obs_>2σ(I_obs_): _refine_ls_R_factor_all = 0.0324, _refine_ls_R_factor_gt = 0.0291, _refine_ls_wR_factor_ref = 0.0710, _refine_ls_wR_factor_gt = 0.0703, 428 parameters, 8981 diffractions, 7 restraints, 196 constraints. However, the refinement with *JANA*2006 did not include 4 × 4 × 0.946 electrons per unit cell into the calculation because the positions of the hydroxy as well as of the methyl hydrogen atoms of propionic acid were not determined.

### Fractional atomic coordinates and isotropic or equivalent isotropic displacement parameters (Å^2^)

**Table:**

	*x*	*y*	*z*	*U*_iso_*/*U*_eq_	Occ. (<1)
Ba1	0.265102 (12)	0.276842 (14)	0.409817 (6)	0.01040 (6)	
O1	0.20473 (14)	0.15684 (17)	0.48047 (7)	0.0141 (7)	
O2	0.20378 (14)	0.31250 (17)	0.49391 (7)	0.0160 (7)	
C1	0.19826 (19)	0.2274 (3)	0.50646 (10)	0.0139 (10)	
C2	0.1819 (2)	0.2043 (3)	0.55457 (10)	0.0194 (11)	
H1c2	0.125429	0.174223	0.557629	0.0233*	
H2c2	0.221038	0.153268	0.564196	0.0233*	
C3	0.1884 (3)	0.2883 (3)	0.58557 (11)	0.0265 (12)	
H1c3	0.170755	0.268526	0.614948	0.0397*	
H2c3	0.247161	0.310864	0.586517	0.0397*	
H3c3	0.151542	0.339958	0.575311	0.0397*	
O3	0.77274 (14)	0.27521 (18)	0.67975 (7)	0.0165 (7)	
O4	0.70280 (16)	0.13907 (18)	0.67159 (8)	0.0216 (8)	
C4	0.7343 (2)	0.2046 (3)	0.69537 (10)	0.0161 (10)	
C5	0.7235 (3)	0.1981 (3)	0.74443 (11)	0.0272 (13)	
H1c5	0.706908	0.132246	0.752502	0.0327*	
H2c5	0.675065	0.238198	0.753611	0.0327*	
C6	0.8025 (3)	0.2269 (3)	0.77026 (11)	0.0310 (13)	
H1c6	0.79028	0.223693	0.801666	0.0465*	
H2c6	0.849043	0.183217	0.763236	0.0465*	
H3c6	0.818705	0.29217	0.762411	0.0465*	
Ba2	0.596864 (11)	0.480169 (14)	0.553971 (6)	0.00925 (6)	
O5	0.39730 (13)	0.31291 (17)	0.46155 (7)	0.0149 (7)	
O6	0.50137 (14)	0.39354 (17)	0.49305 (7)	0.0170 (7)	
C7	0.45768 (19)	0.3181 (2)	0.48840 (9)	0.0124 (9)	
C8	0.4801 (2)	0.2324 (3)	0.51561 (11)	0.0221 (11)	
H1c8	0.519283	0.251628	0.539298	0.0266*	
H2c8	0.511697	0.185977	0.497441	0.0266*	
C9	0.4028 (2)	0.1839 (3)	0.53535 (12)	0.0265 (12)	
H1c9	0.421114	0.132078	0.554706	0.0397*	
H2c9	0.369982	0.230539	0.55218	0.0397*	
H3c9	0.367423	0.15771	0.511864	0.0397*	
O1w	0.69585 (16)	0.53342 (18)	0.47792 (8)	0.0176 (8)	
H1o1w	0.720 (2)	0.4835 (17)	0.4845 (14)	0.0264*	
H2o1w	0.730 (2)	0.575 (2)	0.4853 (13)	0.0264*	
O2w	0.43714 (16)	0.4553 (2)	0.59034 (8)	0.0263 (9)	
H1o2w	0.398 (2)	0.454 (3)	0.5728 (12)	0.0395*	
H2o2w	0.419 (3)	0.472 (3)	0.6140 (8)	0.0395*	
Ba3	0.723298 (12)	0.460242 (14)	0.675708 (6)	0.01063 (6)	
O7	0.32899 (14)	0.09999 (17)	0.39377 (7)	0.0146 (7)	
O8	0.26230 (14)	−0.02799 (17)	0.41819 (8)	0.0147 (7)	
C10	0.3271 (2)	0.0247 (2)	0.41603 (10)	0.0124 (9)	
C11	0.4054 (2)	−0.0038 (3)	0.44141 (13)	0.0280 (13)	
H1c11	0.396235	0.00861	0.472953	0.0336*	
H2c11	0.413213	−0.07378	0.439522	0.0336*	
C12	0.4852 (2)	0.0456 (3)	0.42663 (16)	0.0426 (17)	
H1c12	0.501238	0.022104	0.397576	0.0639*	
H2c12	0.475349	0.114565	0.425252	0.0639*	
H3c12	0.530935	0.03221	0.447435	0.0639*	
O9	0.57946 (15)	0.58185 (18)	0.69864 (7)	0.0184 (7)	
O10	0.61677 (15)	0.58789 (17)	0.62908 (7)	0.0152 (7)	
C13	0.5694 (2)	0.6136 (2)	0.66061 (11)	0.0159 (10)	
C14	0.4989 (2)	0.6848 (3)	0.65013 (13)	0.0311 (13)	
H1c14	0.502517	0.70361	0.618957	0.0373*	
H2c14	0.443144	0.653296	0.653563	0.0373*	
C15	0.5020 (3)	0.7718 (4)	0.67817 (18)	0.0504 (19)	
H1c15	0.459821	0.817969	0.667909	0.0756*	
H2c15	0.489699	0.754313	0.708558	0.0756*	
H3c15	0.558621	0.800339	0.676466	0.0756*	
O11	0.62289 (13)	0.33741 (17)	0.61727 (7)	0.0127 (7)	
O12	0.57444 (14)	0.35474 (18)	0.68509 (7)	0.0177 (7)	
C16	0.5717 (2)	0.3152 (2)	0.64849 (10)	0.0147 (10)	
C17	0.5076 (3)	0.2391 (3)	0.63987 (13)	0.0366 (14)	
H1c17	0.536797	0.177233	0.635543	0.0439*	
H2c17	0.479622	0.251292	0.611423	0.0439*	
C18	0.4407 (4)	0.2285 (4)	0.67540 (16)	0.069 (2)	
H1c18	0.404027	0.174063	0.668575	0.1033*	
H2c18	0.468423	0.217922	0.703673	0.1033*	
H3c18	0.406464	0.286802	0.676826	0.1033*	
Ba4	0.5	0.45639 (2)	0.75	0.01352 (8)	
O13	0.68012 (16)	0.44722 (19)	0.76008 (7)	0.0211 (8)	
O14	0.61403 (16)	0.4787 (2)	0.82236 (8)	0.0269 (9)	
C19	0.6806 (2)	0.4662 (3)	0.80085 (11)	0.0192 (11)	
C20	0.7660 (2)	0.4712 (3)	0.82319 (11)	0.0248 (13)	
H1c20	0.808934	0.494763	0.802197	0.0298*	
H2c20	0.785871	0.405822	0.829933	0.0298*	
C21	0.7687 (2)	0.5316 (3)	0.86451 (12)	0.0225 (12)	
H1c21	0.82734	0.536988	0.874707	0.0338*	
H2c21	0.746258	0.595323	0.858178	0.0338*	
H3c21	0.734129	0.501355	0.887271	0.0338*	
C22	0.4426 (5)	−0.0002 (7)	0.2770 (3)	0.031 (4)	0.473 (4)
C24a	0.508 (2)	0.062 (4)	0.2061 (7)	0.16 (2)	0.304 (15)
C24b	0.533 (2)	−0.014 (2)	0.2077 (7)	0.031 (4)	0.169 (15)
H1c23a	0.552935	0.078307	0.268738	0.0718*	0.304 (15)
H2c23a	0.562725	−0.025336	0.251889	0.0718*	0.304 (15)
H1c23b	0.524842	0.099005	0.249815	0.0718*	0.169 (15)
H2c23b	0.572777	0.014063	0.270039	0.0718*	0.169 (15)
O15	0.3759 (5)	0.0388 (6)	0.2758 (2)	0.050 (3)	0.473 (4)
O16	0.4501 (8)	−0.0790 (10)	0.2993 (4)	0.071 (3)	0.473 (4)
C23	0.5222 (10)	0.0286 (11)	0.2521 (6)	0.060 (5)	0.473 (4)

### Atomic displacement parameters (Å^2^)

**Table:**

	*U*^11^	*U*^22^	*U*^33^	*U*^12^	*U*^13^	*U*^23^
Ba1	0.01365 (10)	0.00916 (11)	0.00838 (10)	−0.00298 (7)	−0.00251 (6)	0.00078 (7)
O1	0.0172 (11)	0.0147 (13)	0.0103 (11)	−0.0051 (10)	−0.0004 (8)	−0.0024 (9)
O2	0.0184 (12)	0.0152 (13)	0.0143 (12)	−0.0012 (10)	0.0011 (9)	0.0008 (9)
C1	0.0091 (15)	0.0207 (19)	0.0118 (16)	−0.0031 (13)	−0.0017 (11)	−0.0001 (13)
C2	0.0243 (19)	0.020 (2)	0.0139 (17)	0.0002 (15)	−0.0026 (13)	0.0002 (13)
C3	0.043 (2)	0.023 (2)	0.0128 (17)	0.0025 (18)	0.0039 (15)	−0.0018 (14)
O3	0.0211 (13)	0.0181 (14)	0.0104 (12)	0.0062 (10)	0.0022 (9)	0.0031 (9)
O4	0.0291 (14)	0.0141 (14)	0.0216 (13)	0.0038 (11)	0.0049 (10)	0.0003 (10)
C4	0.0210 (18)	0.0154 (19)	0.0118 (16)	0.0085 (14)	0.0039 (12)	0.0006 (13)
C5	0.043 (2)	0.025 (2)	0.0132 (18)	−0.0031 (18)	0.0071 (15)	0.0051 (15)
C6	0.049 (3)	0.030 (2)	0.0146 (18)	0.007 (2)	−0.0027 (16)	0.0009 (16)
Ba2	0.00957 (10)	0.00981 (11)	0.00836 (10)	−0.00025 (7)	−0.00123 (6)	−0.00020 (7)
O5	0.0169 (12)	0.0160 (13)	0.0118 (11)	−0.0008 (10)	−0.0045 (8)	0.0012 (9)
O6	0.0163 (12)	0.0137 (13)	0.0211 (12)	−0.0008 (10)	−0.0078 (9)	−0.0027 (9)
C7	0.0123 (15)	0.0139 (17)	0.0110 (15)	0.0021 (13)	0.0009 (11)	−0.0033 (12)
C8	0.0190 (18)	0.018 (2)	0.030 (2)	0.0020 (15)	−0.0033 (14)	0.0085 (15)
C9	0.0220 (19)	0.024 (2)	0.034 (2)	−0.0054 (16)	−0.0079 (15)	0.0145 (17)
O1w	0.0204 (13)	0.0160 (14)	0.0162 (12)	−0.0027 (11)	−0.0002 (10)	−0.0005 (10)
O2w	0.0137 (13)	0.0527 (19)	0.0125 (13)	−0.0020 (12)	0.0019 (9)	0.0017 (12)
Ba3	0.01169 (10)	0.01076 (11)	0.00942 (10)	−0.00040 (7)	0.00187 (6)	−0.00148 (7)
O7	0.0172 (12)	0.0134 (12)	0.0132 (11)	−0.0022 (10)	−0.0025 (9)	0.0022 (9)
O8	0.0138 (12)	0.0120 (13)	0.0183 (12)	0.0010 (9)	0.0025 (9)	−0.0003 (9)
C10	0.0108 (15)	0.0144 (17)	0.0122 (16)	0.0017 (13)	−0.0001 (11)	−0.0040 (12)
C11	0.025 (2)	0.024 (2)	0.035 (2)	0.0055 (17)	−0.0136 (16)	0.0035 (17)
C12	0.014 (2)	0.039 (3)	0.075 (4)	−0.0002 (19)	−0.018 (2)	0.001 (2)
O9	0.0226 (13)	0.0167 (13)	0.0160 (12)	−0.0012 (11)	0.0052 (9)	0.0005 (10)
O10	0.0197 (12)	0.0160 (13)	0.0100 (11)	−0.0027 (10)	0.0002 (9)	−0.0001 (9)
C13	0.0175 (16)	0.0118 (17)	0.0184 (17)	−0.0030 (14)	0.0017 (13)	−0.0014 (13)
C14	0.024 (2)	0.031 (2)	0.038 (2)	0.0067 (18)	0.0017 (16)	−0.0008 (18)
C15	0.036 (3)	0.041 (3)	0.074 (4)	0.014 (2)	−0.008 (2)	−0.016 (3)
O11	0.0121 (11)	0.0153 (13)	0.0106 (11)	0.0013 (9)	0.0032 (8)	0.0008 (8)
O12	0.0168 (12)	0.0208 (14)	0.0154 (12)	−0.0028 (11)	0.0048 (9)	−0.0055 (9)
C16	0.0157 (16)	0.0146 (18)	0.0137 (16)	−0.0009 (14)	0.0046 (12)	−0.0008 (12)
C17	0.040 (2)	0.039 (3)	0.031 (2)	−0.026 (2)	0.0169 (18)	−0.0183 (19)
C18	0.073 (4)	0.071 (4)	0.062 (3)	−0.061 (4)	0.046 (3)	−0.035 (3)
Ba4	0.01414 (14)	0.01790 (16)	0.00851 (13)	0	0.00393 (9)	0
O13	0.0259 (14)	0.0265 (15)	0.0110 (12)	0.0051 (11)	−0.0003 (10)	−0.0023 (10)
O14	0.0211 (13)	0.048 (2)	0.0111 (13)	0.0057 (13)	0.0015 (10)	0.0005 (11)
C19	0.0256 (19)	0.0198 (19)	0.0123 (17)	0.0049 (15)	0.0013 (13)	0.0010 (13)
C20	0.023 (2)	0.037 (3)	0.0148 (19)	0.0069 (17)	−0.0013 (13)	−0.0007 (16)
C21	0.0209 (19)	0.025 (2)	0.0214 (19)	−0.0045 (16)	−0.0028 (14)	−0.0011 (15)
C22	0.037 (7)	0.028 (5)	0.029 (6)	−0.003 (5)	−0.014 (4)	0.006 (4)
C24a	0.12 (3)	0.23 (5)	0.14 (3)	0.01 (3)	0.05 (2)	0.09 (3)
C24b	0.037 (7)	0.028 (5)	0.029 (6)	0.003 (5)	−0.014 (4)	−0.006 (4)
O15	0.038 (4)	0.057 (5)	0.054 (5)	−0.020 (4)	0.014 (3)	−0.015 (4)
O16	0.037 (4)	0.059 (6)	0.116 (7)	0.000 (4)	0.002 (4)	0.046 (5)
C23	0.050 (11)	0.056 (8)	0.074 (9)	−0.014 (6)	−0.031 (9)	0.027 (7)

### Geometric parameters (Å, º)

**Table:**

O1—C1	1.273 (4)	C18—H1c18	0.98
O2—C1	1.256 (4)	C18—H2c18	0.98
C1—C2	1.528 (4)	C18—H3c18	0.98
C2—H1c2	0.99	O13—C19	1.274 (4)
C2—H2c2	0.99	O14—C19	1.251 (4)
C2—C3	1.515 (5)	C19—C20	1.513 (5)
C3—H1c3	0.98	C20—H1c20	0.99
C3—H2c3	0.98	C20—H2c20	0.99
C3—H3c3	0.98	C20—C21	1.521 (5)
O3—C4	1.255 (4)	C21—H1c21	0.98
O4—C4	1.272 (4)	C21—H2c21	0.98
C4—C5	1.512 (5)	C21—H3c21	0.98
C5—H1c5	0.99	C22—O15	1.187 (12)
C5—H2c5	0.99	C22—O16	1.303 (18)
C5—C6	1.529 (6)	C22—C23	1.523 (18)
C6—H1c6	0.98	C24a—C23	1.50 (3)
C6—H2c6	0.98	C24b—C23	1.49 (3)
C6—H3c6	0.98	H1c23a—C23	0.99
O5—C7	1.260 (4)	H2c23a—C23	0.99
O6—C7	1.270 (4)	H1c23b—C23	0.99
C7—C8	1.504 (5)	H2c23b—C23	0.99
C8—H1c8	0.99	Ba1—O1	2.898 (2)
C8—H2c8	0.99	Ba1—O2	2.791 (2)
C8—C9	1.521 (5)	Ba1—O3^i^	2.835 (2)
C9—H1c9	0.98	Ba1—O4^i^	2.923 (2)
C9—H2c9	0.98	Ba1—O5	2.666 (2)
C9—H3c9	0.98	Ba1—O7	2.720 (2)
O1w—H1o1w	0.82 (3)	Ba1—O8^ii^	2.781 (2)
O1w—H2o1w	0.82 (3)	Ba1—O10^iii^	2.913 (2)
O2w—H1o2w	0.82 (3)	Ba1—O11^i^	2.879 (2)
O2w—H2o2w	0.81 (3)	Ba2—O1^iv^	2.774 (2)
O7—C10	1.256 (4)	Ba2—O5^iii^	2.940 (2)
O8—C10	1.263 (4)	Ba2—O6	2.685 (2)
C10—C11	1.513 (5)	Ba2—O6^iii^	2.757 (2)
C11—H1c11	0.99	Ba2—O1w	2.898 (2)
C11—H2c11	0.99	Ba2—O2w	2.777 (3)
C11—C12	1.507 (6)	Ba2—O8^iv^	2.827 (2)
C12—H1c12	0.98	Ba2—O10	2.765 (2)
C12—H2c12	0.98	Ba2—O11	2.813 (2)
C12—H3c12	0.98	Ba3—O3	2.711 (3)
O9—C13	1.255 (4)	Ba3—O4^v^	2.767 (3)
O10—C13	1.272 (4)	Ba3—O7^iv^	2.829 (2)
C13—C14	1.528 (5)	Ba3—O8^iv^	3.084 (2)
C14—H1c14	0.99	Ba3—O9	2.924 (2)
C14—H2c14	0.99	Ba3—O10	2.838 (2)
C14—C15	1.491 (7)	Ba3—O11	2.943 (2)
C15—H1c15	0.98	Ba3—O12	2.791 (2)
C15—H2c15	0.98	Ba3—O13	2.673 (2)
C15—H3c15	0.98	Ba3—O15^iv^	2.828 (7)
H1c15—H2c15	1.6003	Ba4—O9	2.670 (2)
H1c15—H3c15	1.6003	Ba4—O9^vi^	2.670 (2)
H2c15—H3c15	1.6003	Ba4—O12	2.710 (2)
O11—C16	1.288 (4)	Ba4—O12^vi^	2.710 (2)
O12—C16	1.249 (4)	Ba4—O13	2.862 (3)
C16—C17	1.494 (5)	Ba4—O13^vi^	2.862 (3)
C17—H1c17	0.99	Ba4—O14	2.868 (2)
C17—H2c17	0.99	Ba4—O14^vi^	2.868 (2)
C17—C18	1.522 (7)		
			
O1—C1—O2	122.8 (3)	O2—Ba1—O8^ii^	71.65 (7)
O1—C1—C2	116.7 (3)	O2—Ba1—O10^iii^	118.77 (6)
O2—C1—C2	120.5 (3)	O2—Ba1—O11^i^	95.39 (6)
C1—C2—H1c2	109.47	O3^i^—Ba1—O4^i^	45.29 (7)
C1—C2—H2c2	109.47	O3^i^—Ba1—O5	126.01 (6)
C1—C2—C3	115.2 (3)	O3^i^—Ba1—O7	64.90 (7)
H1c2—C2—H2c2	103.07	O3^i^—Ba1—O8^ii^	110.40 (7)
H1c2—C2—C3	109.47	O3^i^—Ba1—O10^iii^	75.30 (6)
H2c2—C2—C3	109.47	O3^i^—Ba1—O11^i^	67.20 (6)
C2—C3—H1c3	109.47	O4^i^—Ba1—O5	133.75 (7)
C2—C3—H2c3	109.47	O4^i^—Ba1—O7	109.79 (7)
C2—C3—H3c3	109.47	O4^i^—Ba1—O8^ii^	68.25 (7)
H1c3—C3—H2c3	109.47	O4^i^—Ba1—O10^iii^	66.77 (7)
H1c3—C3—H3c3	109.47	O4^i^—Ba1—O11^i^	73.57 (7)
H2c3—C3—H3c3	109.47	O5—Ba1—O7	89.40 (7)
O3—C4—O4	122.7 (3)	O5—Ba1—O8^ii^	83.15 (7)
O3—C4—C5	118.6 (3)	O5—Ba1—O10^iii^	67.52 (6)
O4—C4—C5	118.6 (3)	O5—Ba1—O11^i^	152.39 (7)
C4—C5—H1c5	109.47	O7—Ba1—O8^ii^	166.01 (7)
C4—C5—H2c5	109.47	O7—Ba1—O10^iii^	106.39 (7)
C4—C5—C6	113.8 (3)	O7—Ba1—O11^i^	74.36 (6)
H1c5—C5—H2c5	104.72	O8^ii^—Ba1—O10^iii^	59.80 (7)
H1c5—C5—C6	109.47	O8^ii^—Ba1—O11^i^	116.89 (6)
H2c5—C5—C6	109.47	O10^iii^—Ba1—O11^i^	138.06 (6)
C5—C6—H1c6	109.47	O1^iv^—Ba2—O5^iii^	127.04 (6)
C5—C6—H2c6	109.47	O1^iv^—Ba2—O6	76.61 (7)
C5—C6—H3c6	109.47	O1^iv^—Ba2—O6^iii^	126.29 (6)
H1c6—C6—H2c6	109.47	O1^iv^—Ba2—O1w	62.81 (7)
H1c6—C6—H3c6	109.47	O1^iv^—Ba2—O2w	128.49 (8)
H2c6—C6—H3c6	109.47	O1^iv^—Ba2—O8^iv^	73.22 (7)
O5—C7—O6	122.2 (3)	O1^iv^—Ba2—O10	128.55 (7)
O5—C7—C8	119.4 (3)	O1^iv^—Ba2—O11	71.28 (6)
O6—C7—C8	118.4 (3)	O5^iii^—Ba2—O6	110.59 (7)
C7—C8—H1c8	109.47	O5^iii^—Ba2—O6^iii^	45.60 (6)
C7—C8—H2c8	109.47	O5^iii^—Ba2—O1w	66.41 (7)
C7—C8—C9	112.8 (3)	O5^iii^—Ba2—O2w	102.51 (8)
H1c8—C8—H2c8	105.89	O5^iii^—Ba2—O8^iv^	77.63 (6)
H1c8—C8—C9	109.47	O5^iii^—Ba2—O10	65.91 (6)
H2c8—C8—C9	109.47	O5^iii^—Ba2—O11	143.89 (6)
C8—C9—H1c9	109.47	O6—Ba2—O6^iii^	67.25 (7)
C8—C9—H2c9	109.47	O6—Ba2—O1w	82.13 (7)
C8—C9—H3c9	109.47	O6—Ba2—O2w	73.22 (7)
H1c9—C9—H2c9	109.47	O6—Ba2—O8^iv^	146.54 (7)
H1c9—C9—H3c9	109.47	O6—Ba2—O10	152.38 (7)
H2c9—C9—H3c9	109.47	O6—Ba2—O11	103.72 (7)
H1o1w—O1w—H2o1w	104 (3)	O6^iii^—Ba2—O1w	73.74 (7)
H1o2w—O2w—H2o2w	109 (4)	O6^iii^—Ba2—O2w	77.20 (7)
O7—C10—O8	122.6 (3)	O6^iii^—Ba2—O8^iv^	121.61 (7)
O7—C10—C11	118.7 (3)	O6^iii^—Ba2—O10	98.40 (7)
O8—C10—C11	118.7 (3)	O6^iii^—Ba2—O11	153.77 (6)
C10—C11—H1c11	109.47	O1w—Ba2—O2w	147.42 (7)
C10—C11—H2c11	109.47	O1w—Ba2—O8^iv^	71.48 (7)
C10—C11—C12	114.1 (3)	O1w—Ba2—O10	117.64 (7)
H1c11—C11—H2c11	104.42	O1w—Ba2—O11	130.99 (7)
H1c11—C11—C12	109.47	O2w—Ba2—O8^iv^	138.40 (7)
H2c11—C11—C12	109.47	O2w—Ba2—O10	80.75 (7)
C11—C12—H1c12	109.47	O2w—Ba2—O11	76.60 (7)
C11—C12—H2c12	109.47	O8^iv^—Ba2—O10	61.04 (7)
C11—C12—H3c12	109.47	O8^iv^—Ba2—O11	80.03 (6)
H1c12—C12—H2c12	109.47	O10—Ba2—O11	78.51 (6)
H1c12—C12—H3c12	109.47	O3—Ba3—O4^v^	138.34 (7)
H2c12—C12—H3c12	109.47	O3—Ba3—O7^iv^	65.10 (7)
O9—C13—O10	121.8 (3)	O3—Ba3—O8^iv^	106.22 (6)
O9—C13—C14	121.2 (3)	O3—Ba3—O9	140.37 (7)
O10—C13—C14	117.0 (3)	O3—Ba3—O10	142.77 (7)
C13—C14—H1c14	109.47	O3—Ba3—O11	67.87 (6)
C13—C14—H2c14	109.47	O3—Ba3—O12	74.39 (7)
C13—C14—C15	112.9 (3)	O3—Ba3—O13	87.95 (7)
H1c14—C14—H2c14	105.79	O3—Ba3—O15^iv^	74.66 (16)
H1c14—C14—C15	109.47	O4^v^—Ba3—O7^iv^	89.29 (7)
H2c14—C14—C15	109.47	O4^v^—Ba3—O8^iv^	66.10 (6)
C14—C15—H1c15	109.47	O4^v^—Ba3—O9	79.07 (7)
C14—C15—H2c15	109.47	O4^v^—Ba3—O10	69.89 (7)
C14—C15—H3c15	109.47	O4^v^—Ba3—O11	136.80 (6)
H1c15—C15—H2c15	109.47	O4^v^—Ba3—O12	147.07 (7)
H1c15—C15—H3c15	109.47	O4^v^—Ba3—O13	102.31 (7)
H2c15—C15—H3c15	109.47	O4^v^—Ba3—O15^iv^	70.16 (16)
O11—C16—O12	122.3 (3)	O7^iv^—Ba3—O8^iv^	43.61 (6)
O11—C16—C17	117.8 (3)	O7^iv^—Ba3—O9	144.26 (6)
O12—C16—C17	119.9 (3)	O7^iv^—Ba3—O10	99.24 (6)
C16—C17—H1c17	109.47	O7^iv^—Ba3—O11	71.80 (6)
C16—C17—H2c17	109.47	O7^iv^—Ba3—O12	114.55 (7)
C16—C17—C18	114.4 (4)	O7^iv^—Ba3—O13	148.63 (7)
H1c17—C17—H2c17	104.01	O7^iv^—Ba3—O15^iv^	83.84 (15)
H1c17—C17—C18	109.47	O8^iv^—Ba3—O9	101.45 (6)
H2c17—C17—C18	109.47	O8^iv^—Ba3—O10	57.13 (6)
C17—C18—H1c18	109.47	O8^iv^—Ba3—O11	73.92 (6)
C17—C18—H2c18	109.47	O8^iv^—Ba3—O12	115.25 (6)
C17—C18—H3c18	109.47	O8^iv^—Ba3—O13	165.74 (7)
H1c18—C18—H2c18	109.47	O8^iv^—Ba3—O15^iv^	108.43 (15)
H1c18—C18—H3c18	109.47	O9—Ba3—O10	45.02 (6)
H2c18—C18—H3c18	109.47	O9—Ba3—O11	93.91 (6)
O13—C19—O14	122.6 (3)	O9—Ba3—O12	68.32 (7)
O13—C19—C20	117.1 (3)	O9—Ba3—O13	67.09 (7)
O14—C19—C20	120.3 (3)	O9—Ba3—O15^iv^	122.20 (16)
C19—C20—H1c20	109.47	O10—Ba3—O11	75.23 (6)
C19—C20—H2c20	109.47	O10—Ba3—O12	83.50 (7)
C19—C20—C21	115.2 (3)	O10—Ba3—O13	112.10 (7)
H1c20—C20—H2c20	103.1	O10—Ba3—O15^iv^	139.87 (16)
H1c20—C20—C21	109.47	O11—Ba3—O12	45.51 (6)
H2c20—C20—C21	109.47	O11—Ba3—O13	114.08 (7)
C20—C21—H1c21	109.47	O11—Ba3—O15^iv^	141.20 (16)
C20—C21—H2c21	109.47	O12—Ba3—O13	69.52 (7)
C20—C21—H3c21	109.47	O12—Ba3—O15^iv^	131.73 (16)
H1c21—C21—H2c21	109.47	O13—Ba3—O15^iv^	73.25 (16)
H1c21—C21—H3c21	109.47	O9—Ba4—O9^vi^	97.61 (7)
H2c21—C21—H3c21	109.47	O9—Ba4—O12	73.28 (7)
O15—C22—O16	119.1 (10)	O9—Ba4—O12^vi^	168.56 (7)
O15—C22—C23	126.5 (11)	O9—Ba4—O13	68.05 (7)
O16—C22—C23	114.3 (10)	O9—Ba4—O13^vi^	115.65 (7)
C22—C23—C24a	115.3 (17)	O9—Ba4—O14	94.96 (7)
C22—C23—C24b	116.0 (17)	O9—Ba4—O14^vi^	76.68 (7)
C22—C23—H1c23a	109.47	O9^vi^—Ba4—O12	168.56 (7)
C22—C23—H2c23a	109.47	O9^vi^—Ba4—O12^vi^	73.28 (7)
C22—C23—H1c23b	109.47	O9^vi^—Ba4—O13	115.65 (7)
C22—C23—H2c23b	109.47	O9^vi^—Ba4—O13^vi^	68.05 (7)
C24a—C23—H1c23a	109.47	O9^vi^—Ba4—O14	76.68 (7)
C24a—C23—H2c23a	109.47	O9^vi^—Ba4—O14^vi^	94.96 (7)
C24b—C23—H1c23b	109.47	O12—Ba4—O12^vi^	116.58 (7)
C24b—C23—H2c23b	109.47	O12—Ba4—O13	67.95 (7)
H1c23a—C23—H2c23a	102.92	O12—Ba4—O13^vi^	109.16 (7)
H1c23b—C23—H2c23b	102.1	O12—Ba4—O14	110.46 (7)
O1—Ba1—O2	45.88 (7)	O12—Ba4—O14^vi^	76.42 (7)
O1—Ba1—O3^i^	125.73 (7)	O12^vi^—Ba4—O13	109.16 (7)
O1—Ba1—O4^i^	139.24 (7)	O12^vi^—Ba4—O13^vi^	67.95 (7)
O1—Ba1—O5	85.73 (6)	O12^vi^—Ba4—O14	76.42 (7)
O1—Ba1—O7	74.18 (6)	O12^vi^—Ba4—O14^vi^	110.46 (7)
O1—Ba1—O8^ii^	116.81 (7)	O13—Ba4—O13^vi^	174.86 (7)
O1—Ba1—O10^iii^	153.16 (6)	O13—Ba4—O14	45.47 (7)
O1—Ba1—O11^i^	68.60 (6)	O13—Ba4—O14^vi^	135.33 (7)
O2—Ba1—O3^i^	161.74 (7)	O13^vi^—Ba4—O14	135.33 (7)
O2—Ba1—O4^i^	126.49 (7)	O13^vi^—Ba4—O14^vi^	45.47 (7)
O2—Ba1—O5	72.03 (6)	O14—Ba4—O14^vi^	167.46 (9)
O2—Ba1—O7	117.23 (7)		

Symmetry codes: (i) *x*−1/2, −*y*+1/2, −*z*+1; (ii) −*x*+1/2, *y*+1/2, *z*; (iii) −*x*+1, −*y*+1, −*z*+1; (iv) *x*+1/2, −*y*+1/2, −*z*+1; (v) −*x*+3/2, *y*+1/2, *z*; (vi) −*x*+1, *y*, −*z*+3/2.

### Hydrogen-bond geometry (Å, º)

**Table:**

*D*—H···*A*	*D*—H	H···*A*	*D*···*A*	*D*—H···*A*
O1*w*—H1*o*1*w*···O1^iv^	0.82 (3)	2.25 (3)	2.957 (3)	145 (4)
O1*w*—H2*o*1*w*···O2^iii^	0.82 (3)	2.00 (3)	2.813 (3)	171 (3)
O2*w*—H1*o*2*w*···O1*w*^iii^	0.82 (3)	2.15 (3)	2.963 (3)	172 (4)
O2*w*—H2*o*2*w*···O14^vi^	0.81 (3)	2.01 (3)	2.807 (3)	164 (4)
C17—H1*c*17···O16^vii^	0.99	2.43	2.989 (15)	115.22

Symmetry codes: (iii) −*x*+1, −*y*+1, −*z*+1; (iv) *x*+1/2, −*y*+1/2, −*z*+1; (vi) −*x*+1, *y*, −*z*+3/2; (vii) −*x*+1, −*y*, −*z*+1.

## References

[bb1] Brandenburg, K. (2005). *DIAMOND*. Crystal Impact GbR, Postfach 1251, D-53002 Bonn, Germany.

[bb2] Brese, N. E. & O’Keeffe, M. (1991). *Acta Cryst.* B**47**, 192–197.

[bb3] Desiraju, G. & Steiner, T. (1999). *The Weak Hydrogen Bond*, p. 65. Oxford University Press.

[bb4] Fábry, J. (2020). Unpublished results.

[bb5] Gesi, K. (1993). *J. Phys. Soc. Jpn*, **62**, 4511–4515.

[bb6] Gilli, G. & Gilli, P. (2009). *The Nature of the Hydrogen Bond*, p. 61. New York: Oxford University Press.

[bb7] Groom, C. R., Bruno, I. J., Lightfoot, M. P. & Ward, S. C. (2016). *Acta Cryst.* B**72**, 171–179. 10.1107/S2052520616003954 PMC4822653 27048719

[bb8] International Centre for Diffraction Data (2019). PDF-4 (Powder Diffraction File 4).

[bb9] Nakamura, E. & Deguchi, K. (1992). *Ferroelectrics*, **137**, 153–163.

[bb10] *Origin* (2000). OriginLab Corporation, USA.

[bb11] Petříček, V., Dušek, M. & Palatinus, L. (2014). *Z. Kristallogr.* **229**, 345–352.

[bb12] Rigaku OD (2019). *CrysAlis PRO*. Rigaku Oxford Diffraction, Yarnton, England.

[bb13] Sheldrick, G. M. (2015*a*). *Acta Cryst.* A**71**, 3–8.

[bb14] Sheldrick, G. M. (2015*b*). *Acta Cryst.* C**71**, 3–8.

[bb15] Spek, A. L. (2015). *Acta Cryst.* C**71**, 9–18. 10.1107/S2053229614024929 25567569

[bb16] Stadnicka, K. & Glazer, A. M. (1980). *Acta Cryst.* B**36**, 2977–2985.

